# Screen to Intervene; establishing a dedicated metabolic clinic for patients with chronic mental illness in an Irish Metal Health Service

**DOI:** 10.1192/j.eurpsy.2022.521

**Published:** 2022-09-01

**Authors:** M. Usman, F. Saleem, B. Mccafferty, J.H.P. Tan, M. Zubir, D. Adamis

**Affiliations:** 1 St James’s Hospital Dublin, Liaison Psychiatry For Elderly, Dublin, Ireland; 2 Mayo University Hospital, Psychiatry, Castlebar, Ireland; 3 Bayview Family Practice, Bundoran, Donegal., General Practice, Bundoran, Ireland; 4 Maudsley Hospital, Psychiatry, South London & Maudsley Nhs Foundation Trust, London, United Kingdom; 5 The Rotunda Hospital, Specialist Perinatal Mental Health Service, Dublin, Ireland; 6 Sligo Leitrim Mental Health Services, St. Columba’s Hospital,, Psychiatry, Sligo, Ireland

**Keywords:** Metabolic, chronic mental illness, physical health

## Abstract

**Introduction:**

People with serious mental illness exhibit higher morbidity and mortality rates of chronic diseases than the general population.

**Objectives:**

The aim of this study was to establish a dedicated clinic for patients with chronic mental illness to monitor physical health in accordance with best practice guidelines.

**Methods:**

Patients were invited to attend the metabolic clinic. The following areas were examined: Personal and family history of cardiovascular disease, diet, exercise, smoking. Mental state examination, waist circumference, BP, pulse, ECG and BMI. Laboratory tests including U+E, LFTs, HbA1c, Lipid profile and other tests as appropriate such as serum lithium. AIMS scale, HoNOS and WHOQOL-BREF scales as additional indicators of global health.

**Results:**

A total of 80 patients attended during 3.5 years of clinic. Mean age was 54.9 years (SD:13.81) at first contact and 45% were females. Mean years in the service was 19.66 (SD:11.54) and mean number of previous hospital admissions was 4.4 (SD:5.63). Metabolic syndrome was present in 42% at first assessment and 20% had at least one new physical abnormality identified during the clinic. A statistically significant difference was found for the psychological domain of the WHOQOL-BREF and the HoNOs particularly at third assessment. (*β*=4.64, Wald x^2^=7.38, df:1, p=0.007, CI:1.3-8.1, *β* =-.889, Wald x^2^=4.08, df:1, p=0.043, CI: -1.752 to-.026) respectively.

**Conclusions:**

The results show a high prevalence of physical health conditions in this cohort, some of which represent a new diagnosis. This implicates better allocation of existing resources for screening and early detection, and potential to run joint clinics with primary care.

**Disclosure:**

No significant relationships.

